# Lack of Association of *Interferon Regulatory Factor 1* with Severe Malaria in Affected Child-Parental Trio Studies across Three African Populations

**DOI:** 10.1371/journal.pone.0004206

**Published:** 2009-01-15

**Authors:** Valentina D. Mangano, Taane G. Clark, Sarah Auburn, Susana Campino, Mahamadou Diakite, Andrew E. Fry, Angela Green, Anna Richardson, Muminatou Jallow, Fatou Sisay-Joof, Margaret Pinder, Michael J. Griffiths, Charles Newton, Norbert Peshu, Thomas N. Williams, Kevin Marsh, Malcolm E. Molyneux, Terrie E. Taylor, David Modiano, Dominic P. Kwiatkowski, Kirk A. Rockett

**Affiliations:** 1 Childhood Infection Group, The Wellcome Trust Centre for Human Genetics, Oxford, United Kingdom; 2 Department of Public Health Sciences, Section of Parasitology, University of Rome La Sapienza, Rome, Italy; 3 Wellcome Trust Sanger Institute, Hinxton, United Kingdom; 4 Medical Research Council, Fajara, The Gambia; 5 Kenya Medical Research Institute Centre for Geographical Medicine Research (Coast), Kilifi, Kenya; 6 Nuffield Department of Medicine, John Radcliffe Hospital, Oxford, United Kingdom; 7 The Malawi–Liverpool–Wellcome Trust Programme of Clinical Tropical Research, Blantyre Malaria Project, College of Medicine, Blantyre, Malawi; 8 Liverpool School of Tropical Medicine, Liverpool, United Kingdom; 9 Department of Internal Medicine, College of Osteopathic Medicine, Michigan State University, East Lansing, Michigan, United Kingdom; London School of Hygiene & Tropical Medicine, United Kingdom

## Abstract

Interferon Regulatory Factor 1 (IRF-1) is a member of the IRF family of transcription factors, which have key and diverse roles in the gene-regulatory networks of the immune system. IRF-1 has been described as a critical mediator of IFN-gamma signalling and as the major player in driving TH1 type responses. It is therefore likely to be crucial in both innate and adaptive responses against intracellular pathogens such as *Plasmodium falciparum.* Polymorphisms at the human *IRF1* locus have been previously found to be associated with the ability to control *P. falciparum* infection in populations naturally exposed to malaria. In order to test whether genetic variation at the *IRF1* locus also affects the risk of developing severe malaria, we performed a family-based test of association for 18 Single Nucleotide Polymorphisms (SNPs) across the gene in three African populations, using genotype data from 961 trios consisting of one affected child and his/her two parents (555 from The Gambia, 204 from Kenya and 202 from Malawi). No significant association with severe malaria or severe malaria subphenotypes (cerebral malaria and severe malaria anaemia) was observed for any of the SNPs/haplotypes tested in any of the study populations. Our results offer no evidence that the molecular pathways regulated by the transcription factor IRF-1 are involved in the immune-based pathogenesis of severe malaria.

## Introduction

Severe malaria is a life-threatening disease that kills over a million individuals every year, with 90% of the deaths occurring in Sub-Sahara African children below the age of five [1]. People living in an endemic area often become infected with *Plasmodium falciparum* malaria during childhood, but a small proportion of children experience severe complications, the clinical outcome depending on many factors, including the genetic make-up of the human host. Malaria is recognised as a very strong selective force in the recent evolutionary history of the human genome [2] and therefore is a candidate of great interest for genetic association analysis. Association studies can inform us on mechanisms of protective immunity against malaria at every stage of infection, the understanding of which is crucial for the development of effective vaccines.

Members of the Interferon Regulatory Factor (IRF) family of transcription factors have key and diverse roles in the gene-regulatory networks of the immune system [3], [4], [5]. In particular IRF-1 has been described as a critical mediator of IFN-gamma signalling and as the major player in driving TH1 type responses [3]. It is therefore likely to be crucial in both innate and adaptive responses against intracellular pathogens such as *P. falciparum.*

In two recent complementary association studies conducted in rural communities and malaria patients of Burkina Faso, we found that *IRF1* polymorphisms were associated with the ability to control *P. falciparum* infection: in adult asymptomatic individuals living in rural villages *IRF-1* alleles were associated with a higher prevalence of *P. falciparum* infection; the same alleles were associated with a higher parasite density in children with uncomplicated and severe malaria [6]. However, our work did not provide conclusive evidence for a role of this locus contributing to or providing protection from the severe manifestations of malaria disease [6], as may be found for a well known genetic factor such as haemoglobin S (HbS) [7].

In order to investigate whether genetic variation at the *IRF1* locus contributes to a child's risk of developing severe malaria, we conducted a large multi-centre association study. As human populations in Africa show a great genetic diversity [8] population structure must be taken into account when conducting population based genetic association studies since it might lead to false positive results [9]. To avoid this drawback we applied the complementary Transmission Disequilibrium Test (TDT) approach [10]. Genotype data were collected from family trios consisting of one affected child and his/her two parents, and the genotype distribution observed in the severe malaria cases was compared to its expected distribution derived on Mendel's law of segregation. A comparison of such family-based association methods versus case-control approaches has been carried out using the well-established association of the sickle cell trait with protection against severe malaria. The two methods were found to have similar power and to give similar estimates of the level of protection [7]. Epidemiological studies have suggested that features of severe malaria, with regards to both age-incidence profiles and clinical spectrum, vary with levels of *P. falciparum* transmission intensity [11]. We therefore conducted our association analysis in trios from three sub-Saharan African countries covering a range of malaria ecologies, The Gambia, Kenya and Malawi.

Here we assess the role of *IRF1* genetic variation in severe malaria, and by considering multiple populations, we able to investigate regional differences, whilst standardizing phenotype definition and study design.

## Results

**Figure 1:**
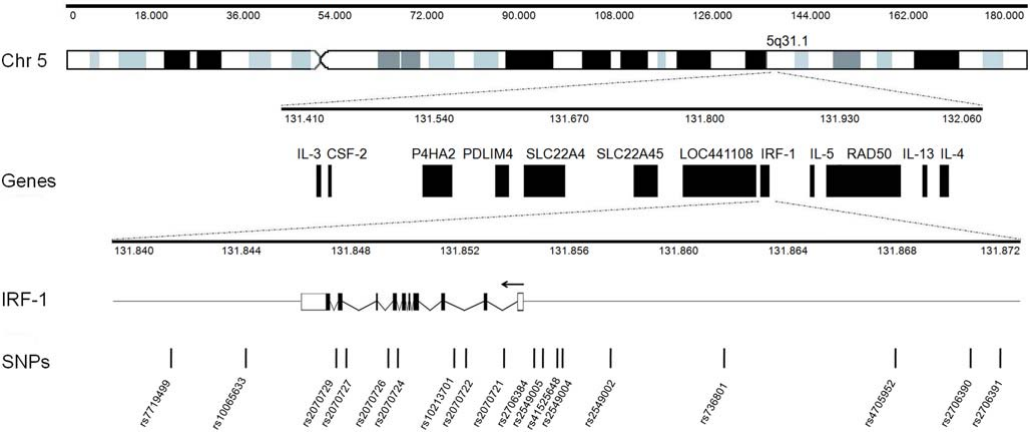
The *IRF1* locus and Single Nucleotide Polymorphisms (SNPs) tested for association with severe malaria in child-parental trios. From top to bottom, the figure shows: human chromosome 5 with its G-banding patterns (black, grey and black bands) where the location of the 5q31.1 band is indicated; genes contained within a 642 kb segment of the 5q31.1 band known as the “Th2 cytokine cluster”; *IRF1* gene region, where the arrow indicates the direction of transcription, horizontal lines indicate intergenic regions, white boxes indicate 5′ and 3′ un-transcribed regions (UTR), black boxes indicate exons and diagonal lines indicate introns; SNPs that were tested for severe malaria in our study populations and their location with respect to the *IRF1* gene region. Coordinates quoted (Mb) are based on Ensembl release 40.

We have genotyped 18 Single Nucleotide Polymorphisms (SNPs) in the *IRF1* genetic region (Figure 1) in 961 nuclear trios comprising one child affected by severe malaria and his/her two parents. Severe malaria patient samples were collected as part of ongoing epidemiological studies at the Royal Victoria Hospital, Banjul, The Gambia (555 trios); the Queen Elizabeth Central Hospital, Blantyre, Malawi (202 trios); and Kilifi District Hospital, Kilifi, Kenya (204 trios). The family trios were assessed for pedigree misspecification using a panel of 48 additional markers spread across the genome and the *Nucl3ar* software package [12].

The allele frequencies calculated among the trio founders (Table 1) differ for many markers between The Gambia and either Kenya or Malawi (significant difference for 14 and 12 SNPs respectively), while they show a greater similarity between the two East-African countries (significant difference for 3 SNPs only), as we could expect on the basis of the geographical distribution and history of the three populations.

**Table 1:** Minor allele frequency of *HBB* and *IRF1* Single Nucleotide Polymorphisms (SNPs) in trio parents from The Gambia, Kenya and Malawi.

SNP	Chr coord	Location	Alleles	The Gambia		Kenya		Malawi		MAF comparison (P-values)		
				MAF	StE	MAF	StE	MAF	StE	G vs K	G vs M	K vs M
HbS (rs334)	11:5204808	exonic	T (A)	0.045	0.006	0.050	0.011	0.004	0.003	0.631	9E-08	3E-08
rs7719499	5:131841990	3′ down	G (C)	0.502	0.015	0.394	0.024	0.395	0.025	2E-07	3E-07	0.992
rs10065633	5:131844615	intronic	T (C)	0.500	0.015	0.394	0.024	0.397	0.025	3E-07	1E-06	0.943
rs2070729	5:131847820	intronic	C (A)	0.385	0.015	0.281	0.022	0.216	0.021	2E-07	1E-17	0.003
rs2070727	5:131848174	intronic	C (A)	0.502	0.015	0.396	0.024	0.395	0.025	3E-07	3E-07	0.992
rs2070726	5:131849637	intronic	C (A)	0.504	0.015	0.394	0.024	0.393	0.025	1E-07	1E-07	0.992
rs2070724	5:131849971	intronic	G (A)	0.485	0.015	0.396	0.024	0.395	0.025	2E-05	2E-05	0.992
rs10213701	5:131851963	intronic	T (A)	0.364	0.015	0.454	0.025	0.476	0.026	1E-05	8E-08	0.415
rs2070722	5:131852385	intronic	T (G)	0.503	0.015	0.393	0.024	0.395	0.025	1E-07	3E-07	0.976
rs2070721	5:131853741	intronic	A (C)	0.388	0.015	0.279	0.024	0.208	0.021	2E-07	2E-19	0.002
rs2706384	5:131854779	5′ up	A (C)	0.404	0.015	0.358	0.025	0.373	0.024	0.029	1E-01	0.576
rs2549005	5:131855090	5′ up	G (A)	0.490	0.015	0.483	0.025	0.469	0.026	0.771	3E-01	0.620
rs41525648	5:131855674	5′ up	C (T)	0.280	0.014	0.238	0.021	0.170	0.019	0.024	1E-09	0.001
rs2549004	5:131855724	5′ up	C (G)	0.404	0.015	0.366	0.024	0.375	0.024	0.067	2E-01	0.749
rs2549002	5:131857477	5′ up	T (G)	0.380	0.015	0.337	0.024	0.350	0.024	0.034	1E-01	0.620
rs736801	5:131861498	intergenic	T (C)	0.054	0.007	0.013	0.006	0.006	0.004	5E-06	2E-08	0.253
rs4705952	5:131867517	intergenic	A (G)	0.354	0.015	0.373	0.024	0.381	0.024	0.358	2E-01	0.781
rs2706390	5:131870179	intergenic	A (C)	0.201	0.012	0.118	0.016	0.094	0.015	2E-07	2E-11	0.141
rs2706391	5:131871205	intergenic	C (T)	0.051	0.007	0.041	0.010	0.053	0.011	0.303	9E-01	0.315

The table shows the SNPs that have been genotyped in the affected child parental trio studies and their frequency in trio parents from The Gambia, Kenya and Malawi. These are not unbiased estimates of the population frequencies. Chr coord is the SNP chromosome coordinate (chromosome: base pair) based on Ensemble release 40. Location: location of the SNP with respect to the *HBB* or *IRF1* locus. Alleles: minor and major alleles (major allele is shown in brackets). MAF: Minor Allele Frequency in pedigree parents (The Gambia, N = 1100; Kenya, N = 408; Malawi, N = 404). StE: Standard Error of the MAF. MAF comparison: P-values of Yates corrected χ^2^ Test for comparison of MAF between populations; a P-value<0.05 is considered statistically significant. G: The Gambia. K: Kenya. M: Malawi.

The power of a TDT analysis to detect transmission distortion is a function of the number of heterozygous parents in the sample and the level of distortion at the locus [13]. Trios in which at least one parent is heterozygous are informative in TDT analysis, and therefore the heterozygosity of a locus can be used as a measure of its information content in family-based association analysis. The observed heterozygosity (H) in the study populations is relatively high: The Gambia, H_G_ = 40.8%; Kenya, H_K_ = 38.7%; Malawi, H_M_ = 38.0%. For comparison, in the HapMap populations Yoruba (Ibadan, Nigeria) and CEPH (Utah residents with ancestry from northern and western Europe) the heterozygosity of the *IRF1* gene is the highest among the Th2 cluster (H_Y_ = 38% and H_C_ = 45%) while the heterozygosity of the neighbouring *IL5* gene is the lowest among the Th2 cluster (H_Y_ = 25% and H_C_ = 21%) (HapMap data release 3.1).

We conducted single marker and haplotype TDT association analysis using a custom script implemented in R statistical software and based on Transmit [14], and the equivalent FBAT approach [15] for comparison of the results. Considering the differences in allele frequencies observed, along with possible differences in clinical spectrum, we conducted the association analysis separately for each of the study populations. When a similar trend was observed in each population, we would pool the data adjusting for study region to increase the power.

The application of the single locus TDT to the 18 *IRF1* SNPs revealed no evidence of association (all P-values>0.05) across all populations, neither with severe malaria or with the sub-phenotypes of cerebral malaria (CM) and severe malaria anaemia (SA) under any of the models tested (additive, dominant, recessive and heterozygous advantage). The results for the test of association with severe malaria under the additive model are shown in Table 2. There was no evidence of heterogeneity or differences in the allelic odds ratios between the CM and SA phenotypes for each SNP (all P-values>0.05), supporting the interpretation above that there were no strong SNP effects being masked by sub-clinical phenotype.

**Table 2:** Single marker Transmission Disequilibrium Test (TDT) of association with severe malaria in Gambian, Kenyan and Malawian child-parental trios.

SNP	Inform	The Gambia				Inform	Kenya				Inform	Malawi			
		OR	LCL	UCL	TDT P		OR	LCL	UCL	TDT P		OR	LCL	UCL	TDT P
HbS (rs334)	170	0.19	0.13	0.28	**6E10^−19^**	26	0.13	0.04	0.43	**9E10^−5^**	3	-	-	-	-
rs7719499	513	0.98	0.82	1.17	0.83	169	0.99	0.73	1.34	0.94	195	0.89	0.67	1.18	0.43
rs10065633	499	0.96	0.80	1.14	0.62	168	1.00	0.74	1.35	1.00	185	0.91	0.68	1.21	0.51
rs2070729	496	1.00	0.84	1.19	1.00	154	1.00	0.73	1.37	1.00	131	0.77	0.55	1.09	0.14
rs2070727	504	0.93	0.78	1.11	0.42	169	0.97	0.71	1.30	0.82	194	0.94	0.71	1.25	0.67
rs2070726	494	0.96	0.81	1.15	0.65	163	1.04	0.76	1.41	0.81	178	0.98	0.73	1.31	0.88
rs2070724	460	0.97	0.81	1.17	0.78	167	1.06	0.78	1.44	0.70	193	1.05	0.79	1.40	0.72
rs10213701	452	0.94	0.78	1.13	0.51	172	1.02	0.76	1.38	0.88	156	0.88	0.64	1.20	0.42
rs2070722	493	0.95	0.80	1.13	0.56	161	1.01	0.74	1.38	0.94	183	0.89	0.66	1.19	0.42
rs2070721	468	1.00	0.83	1.20	1.00	103	1.24	0.84	1.83	0.28	111	0.79	0.54	1.15	0.22
rs2706384	489	0.97	0.81	1.16	0.75	131	0.87	0.62	1.23	0.43	178	0.89	0.67	1.20	0.45
rs2549005	484	1.03	0.86	1.23	0.79	156	0.93	0.68	1.27	0.63	150	0.97	0.71	1.34	0.87
rs41525648	412	0.98	0.81	1.19	0.84	138	1.00	0.72	1.40	1.00	114	0.84	0.58	1.21	0.35
rs2549004	490	0.98	0.82	1.16	0.79	166	0.93	0.69	1.26	0.64	187	0.97	0.73	1.29	0.83
rs2549002	483	0.96	0.80	1.14	0.62	166	0.95	0.70	1.29	0.76	180	0.96	0.71	1.28	0.77
rs736801	85	0.73	0.48	1.13	0.16	9	0.80	0.21	2.98	0.74	4	0.33	0.03	3.20	0.32
rs4705952	462	1.00	0.83	1.20	1.00	191	1.20	0.90	1.59	0.22	177	1.08	0.81	1.45	0.60
rs2706390	313	0.97	0.78	1.21	0.78	83	0.93	0.60	1.43	0.74	63	1.42	0.86	2.35	0.17
rs2706391	95	0.73	0.48	1.09	0.12	26	1.00	0.46	2.16	1.00	28	1.55	0.72	3.30	0.26

The table shows the SNPs tested in association analysis with severe malaria and results of the TDT in the three populations of affected child-parental trios. Inform is the number of informative trios. OR: Odds Ratio comparing the risk of the minor vs major allele. LCL and UCL: Lower and Upper Confidence Limits of a 95% Confidence Interval respectively. TDT P: P-value for the family-based test of association. P-values<0.05 are shown in bold.

In comparison, the HbS allele shows, as expected, a very strong protective effect against severe malaria, with a reduction in risk of 81% and 87% in the Gambian (P = 6×10^−19^) and Kenyan (P = 9×10^−5^) populations, respectively. The effect of the HbS allele could not be tested in the trios from Malawi, due to its low allele frequency in this population and therefore the number of informative trios is not sufficient to compute the statistic.

**Figure 2:**
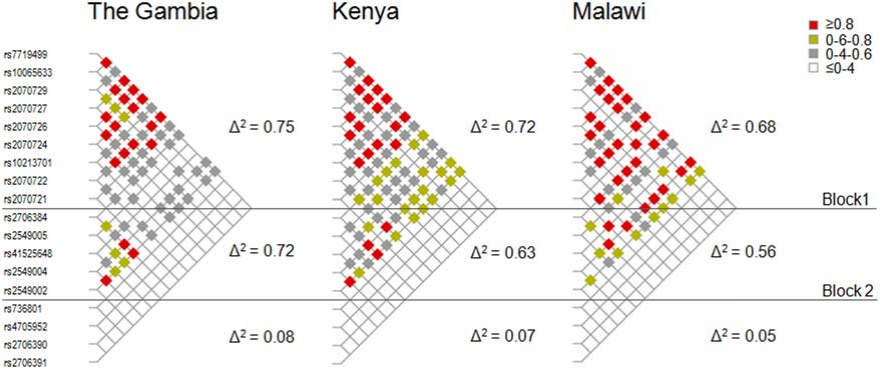
Linkage Disequilibrium (LD ) architecture of the *IRF1* locus in The Gambia, Kenya and Malawi. Typed single nucleotide polymorphisms (SNPs) are represented on the vertical axis and ordered by chromosome position as in Table 1. Diagonal lines in two directions link each SNP to each of the other 17 SNPs. The LD (Δ^2^) between each pair of markers is represented by diamonds of different colours (see legend). Δ^2^ is a simple measure of correlation between markers and is calculated as follows: (f_AB_ f_ab_−f_Ab_ f_aB_)^2^/(f_A_ f_a_ f_B_ f_b_), where f stands for frequency, A and a denote the alleles at the first marker, B and b denote the alleles at the second marker, Ab is the haplotype carrying the major allele for the first marker and the minor allele for the second marker and so on. Blocks 1 and 2 are set of markers in high LD identified within the gene using the HAPLOVIEW application [16].

Linkage Disequilibrium (LD) analysis was performed using the HAPLOVIEW application [16] and revealed a similar haplotype architecture in the three populations (Figure 2). Two blocks of high LD have been identified- the first corresponding to the downstream and coding region (SNPs rs7719499 to rs2070721; average r2: Gambia = 0.75, Kenya = 0.72, Malawi = 0.68) of the gene, the second to the proximal upstream region (<5 kb from the first exon, SNPs rs2706384 to rs2549002; average r2: Gambia = 0.70, Kenya = 0.63, Malawi = 0.56)–while very low levels of LD were observed in the intergenic upstream region (>5 kb from the first exon, SNPs rs736801 to rs2706391; average r2: Gambia = 0.08, Kenya = 0.07, Malawi = 0.05). These results are consistent with previous findings in two other West-African populations from Burkina Faso [6].

We tested the common haplotypes (frequency higher than 5%) within each LD block for association with severe malaria phenotypes. Similarly to the single locus TDT, no significant association was observed (all P-values>0.05) in any of the study populations. Table 3 shows the results for the test of association with severe malaria.

**Table 3:** Haplotype Transmission Disequilibrium Test (TDT) of association with severe malaria in Gambian, Kenyan and Malawian child-parental trios.

Haplotypes	The Gambia						Kenya						Malawi					
	Freq	Inform	OR	LCL	UCL	TDT P	Freq	Inform	OR	LCL	UCL	TDT P	Freq	Inform	OR	LCL	UCL	TDT P
*Block 1*																		
GTCCCGATA	0.38	470	0.96	0.82	1.18	0.64	0.27	151.60	0.97	0.72	1.35	0.83	0.21	124.1	1.11	0.73	1.49	0.57
CCAAAATGC	0.36	438	1.09	0.86	1.25	0.35	0.45	186.80	0.98	0.75	1.32	0.90	0.48	179.2	1.13	0.79	1.41	0.43
CCAAAAAGC	0.14	213	0.84	0.71	1.21	0.20	0.16	110.80	1.06	0.71	1.49	0.74	0.13	85.9	0.82	0.60	1.40	0.36
GTACCGATC	0.11	208	1.06	0.78	1.35	0.67	0.11	79.70	1.03	0.65	1.57	0.89	0.18	99.8	0.84	0.63	1.38	0.40
*Block 2*																		
CATGG	0.51	498	1.00	0.84	1.19	0.96	0.52	183.60	0.90	0.72	1.28	0.50	0.52	181.4	1.01	0.75	1.34	0.95
AGCCT	0.28	409	1.03	0.84	1.23	0.74	0.24	136.90	1.01	0.72	1.40	0.97	0.17	117.5	1.18	0.75	1.54	0.38
AGTCT	0.10	185	1.09	0.78	1.38	0.58	0.09	68.80	1.28	0.69	1.79	0.31	0.16	98.4	0.81	0.61	1.35	0.29
CGTGG	0.09	147	0.91	0.69	1.33	0.57	0.11	83.00	1.08	0.67	1.59	0.73	0.10	66.2	0.86	0.58	1.52	0.55

The table shows the common haplotypes (frequency higher than 5%) within each LD block identified in the gene, their frequency and the results of family-based test of association with severe malaria in the three populations of affected child-parental trios. Freq: frequency of the haplotype in trio parents. These are not unbiased estimates of the population frequencies. OR: Odds Ratio comparing the risk of the untransmitted versus the transmitted haplotype. All other abbreviations as in Table 2.

## Discussion

Rodent malaria studies have shown that IFN-gamma is a key inducer of the immune effector mechanisms that are responsible for the control of both pre-erythrocytic and blood-stage malaria infection [reviewed in 17]. Consistent with the mouse model, studies conducted in human populations have shown that malaria induced IFN-gamma production is associated with lower infection rates and resistance to re-infection [18] and with reduced risk of clinical malaria [19]. For these reasons IFN-gamma is considered as a correlate of cellular immunity to malaria and thus used as a marker for vaccines immunogenicity and efficacy [17]. On the other hand, both in mice and humans, an excessive inflammatory response with high systemic levels of IFN-gamma, TNF-alpha, IL-6 and IL-1, has long been correlated with immune-based pathogenesis of severe disease [reviewed in 20]. The tight regulation of IFN-gamma production and signalling appears therefore of great importance in determining susceptibility to malaria.

We have previously investigated the role of the transcription factor IRF-1, a critical effector molecule in IFN-gamma signalling, in susceptibility to malaria by conducting genetic association studies in Burkina Faso based on cross-sectional parasitological surveys and case-control clinical studies. We have shown that *IRF1* polymorphisms are associated with the control of *P. falciparum* infection (parasite prevalence and density), both in healthy adult subjects and in children with uncomplicated and severe malaria. In those studies we did not find conclusive evidence for the involvement of this locus in the severe manifestations of malaria disease. Indeed, only a trend of association with small differential risk of severe disease was suggested [6].

In the present study we investigated whether genetic variation at the *IRF1* locus affects susceptibility to severe malaria by performing a family based association test in nuclear trios consisting of an affected child and his/her two parents from The Gambia, Kenya and Malawi. The trio design is robust to population stratification. We did not observe any significant associations between the SNPs or haplotypes analysed in severe malaria in any of the three study populations.

There are various possible interpretations of these findings. The effects of *IRF1* polymorphisms on severe malaria may be too small to be detected with this sample size, or may affect only a sub-phenotype of severe malaria. It is likely that the genetic basis of susceptibility to malaria is built upon many different protective genes, each individually resulting in small effects [21]. This study lacks power to detect a small effect [13]. For example, consider a situation where we have 555 trios and wish to detect (assuming a false positive rate of 5%) a multiplicative genotypic effect at a SNP with minor allele frequency of 0.2. If the magnitude of the odds ratio to detect is 1.5 (50% increased risk) we would have >90% power. However, if we wish to detect an odds ratio of only 1.2 (20% increased risk), we would have only 42% power, and would require ∼1400 trios to achieve 80% power. These estimates also assume that the SNP genotyped is in perfect linkage disequilibrium (Δ^2^ = 1) with the causal polymorphism. Overall, we find no evidence that *IRF1* is a strong determinant of susceptibility to severe disease in our study populations.

When considering the exceptionally high diversity of the human genome in African populations [8] we must also take into account that different malaria-resistant alleles may have arisen in different populations due to differences in selective pressure and demographic history. This is the case, for example, of HbC, whose prevalence varies greatly between neighbouring countries and even villages [22], [23]. It is therefore possible that *IRF1* functional variants with an effect on malaria susceptibility/resistance exist in the Fula and Mossi populations of Burkina Faso but not in the study populations presented here. Moreover, we cannot rule out that a different pattern of association could be observed in other African populations as a consequence of a different genetic background. Further disease-association studies across Africa and including Burkina Faso will be needed to answer this question.

Finally, it might be possible that the association of *IRF1* polymorphisms with the ability to control *P. falciparum* infection and the lack of association with susceptibility to severe disease actually reflect differences in the molecular mechanisms underlying protective/pathological immune responses at different stages of infection. The gene regulatory networks of the immune system that are activated during malaria infection and that are responsible for parasite killing may differ from those leading to an over-reaction to the parasite that is harmful to the host, depending for example on the molecular triggers from the parasite or on the tissues involved. That this could be the case has been recently suggested by a genome-wide linkage analysis of parasite density and mild malaria in two Senegalese villages. Regions of linkage showed little if any overlap with genes previously described to be associated with severe malaria, while showing an overlap with genes involved in asthma and atopy related traits [24]. Further insights could be gained by conducting fine mapping studies in multiple populations of larger sample size, where both phenotypes, parasite load and disease severity, could be inspected against the same genetic background. However, the data so far available offer no evidence that the molecular pathways regulated by the transcription factor IRF-1 are involved in immune-based pathogenesis of severe malaria.

## Methods

### Child-parental trios

Severe malaria patient samples were collected as part of ongoing epidemiological studies at the Royal Victoria Hospital, Banjul, The Gambia (555 trios); the Kilifi District Hospital, Kilifi, Kenya (204 trios); and the Queen Elizabeth Central Hospital, Blantyre, Malawi (202 trios). Nuclear family trios comprised one affected child and his two parents.

The research protocols used in this study have obtained approval from the relevant ethics committees: the United Kingdom approval was given by the Oxfordshire Clinical Research Ethics Committee (OxREC); the Gambian approval was given by the Gambia Government/Medical Research Council (MRC) Joint Ethics Committee; the Kenyan approval was given by Kenya Medical Research Institute (KEMRI)/National Ethical Review Committee; the Malawi approval was given by the Health Sciences Research Committee and College of Medicine Research Committee (COMRC).

All DNA samples were collected and genotyped following informed consent from participants. Some of the samples for this study were collected with written consent, others with verbal consent. In all cases the advice of the local ethics committee about the appropriate ways of recording consent in the local research context was followed.

The family trios were assessed for pedigree misspecification using a panel of 48 additional markers spread across the genome and the *Nucl3ar* software package [12].

### Severe malaria cases and phenotypes definition

All cases were children admitted to hospital with evidence of *P. falciparum* on blood film and clinical features of severe malaria [25], [26]. We used a Blantyre coma score of ≤2 as a criterion of cerebral malaria (CM), and haemoglobin <5 g/dl or packed cell volume <15% as a criterion of severe malaria anaemia (SA). Some individuals had both CM and SA. Of the severe malaria cases that were not CM or SA by these criteria, most had lesser degrees of coma (Blantyre coma score 3) or anaemia (Hb 5–6 g/dl), or other complications such as respiratory distress.

The sub-clinical phenotype frequencies in the probands were: (i) The Gambia (443 CM, 239 SA, 141 both CM/SA, 14 other), (ii) Kenya (109 CM, 77 SA, 31 CM/SA, 49 other) and (iii) Malawi (195 CM, 52 SA, 49 CM/SA, 4 other).

The median (range) ages of the Gambian, Kenyan and Malawian cases expressed in months were 41 (0-156), 26 (0-121) and 31.5 (2-156), respectively.

### Markers selection, DNA samples preparation and genotyping

Single Nucleotide Polymorphisms (SNPs) in the region 10 kb upstream to 10 kb downstream of the *IRF1* gene were identified from public databases (dbSNP build 126, Ensemble release 40) and the literature.

18 polymorphisms were selected on the basis of the following criteria: validated status by frequency, haplotype tagging SNP (htSNP) in the HapMap Yoruba population, overall coverage of the locus with regular spread (density of 1 SNP every 2 Kb on average), encompassing exonic, intronic, regulatory regions of the gene. Database identifiers and location with respect to the *IRF1* gene of the 18 SNPs are displayed in Figure 1 and listed in Table 1.

Genomic DNA samples underwent whole genome amplification through either Primer Extension Pre-amplification (PEP) [27] or Multiple Displacement Amplification (MDA) [28] before genotyping.

The genotypes for the selected polymorphisms were determined through the SEQUENOM© MassARRAY™ System [29]. One assay (rs839) did not pass quality filters following initial testing (call rate<90% and evidence that the genotypes in the founders deviated from Hardy-Weinberg equilibrium [30] (P<0.001). This SNP has therefore been excluded from any further analysis.

### Haplotypes and Linkage Disequilibrium architecture

Haplotypes have been constructed using family data and the Stephens-Donnelly method [31]. Haplotypes blocks across the genes have been defined using the HAPLOVIEW application [16], based on the method originally described by Gabriel and colleagues [32].

LD metrics and graphs showing LD patterns have been generated using the MARKER (http://www.gmap.net/perl/marker/marker_entry) application. Typed single nucleotide polymorphisms (SNPs) are represented on the vertical axis and ordered by chromosome position as in Figure 1. The linkage disequilibrium between each pair of markers is represented by diamonds of different colours.

### Statistical methods

Allele frequencies have been calculated among pedigree founders and Hardy-Weinberg equilibrium has been tested, using the dedicated software PEDSTATS [33]. These are not unbiased estimates of the population frequencies. Allele distributions between populations have been compared by Yates-corrected χ^2^ test. If contingency tables had less than 5 expected events per cell, Fisher's exact test was used.

Trio association analysis was performed using the transmission disequilibrium test (TDT). This tests for family-based association in the presence of genetic linkage between a SNP and severe malaria [10], and is robust to the presence of population structure. Single locus and haplotype versions of the TDT were implemented using R statistical software based on Transmit [14]. The FBAT application [15] (software available at http://biosun1.harvard.edu/~fbat/fbat.htm) has been used for comparison of the results.

Haemoglobin genotyping was previously performed on all samples included in the study [34] and association of HbS with severe malaria has been used as positive control for the TDT results.

A P-value of 0.05 has been considered as the threshold for statistical significance.
